# Expression of the recombinant C-terminal of the S1 domain and N-terminal of the S2 domain of the spike protein of porcine epidemic diarrhea virus

**DOI:** 10.14202/vetworld.2021.2913-2918

**Published:** 2021-11-12

**Authors:** Jiraporn Sritun, Natnaree Inthong, Siriluk Jala, Sakuna Phatthanakunanan, Khomson Satchasataporn, Kaitkanoke Sirinarumitr, Preeda Lertwatcharasarakul, Theerapol Sirinarumitr

**Affiliations:** 1 Bio-Veterinary Sciences Program, Faculty of Veterinary Medicine, Kasetsart University, 50 Ngamwongwan Road, Chatuchak, Bangkok, 10900, Thailand; 2 Department of Veterinary Technology, Faculty of Veterinary Technology, Kasetsart University, 50 Ngamwongwan Road, Chatuchak, Bangkok, 10900, Thailand; 3 Kamphaeng Saen Veterinary Diagnosis Center, Faculty of Veterinary Medicine, Kasetsart University, Nakhon Pathom, 73140, Thailand; 4 Department of Companion Animal Clinical Sciences, Faculty of Veterinary Medicine, Kasetsart University, 50 Ngamwongwan Road, Chatuchak, Bangkok, 10900, Thailand; 5 Department of Pathology, Faculty of Veterinary Medicine, Kasetsart University, 50 Ngamwongwan Road, Chatuchak, Bangkok, 10900, Thailand

**Keywords:** diagnostics, genogroup, porcine epidemic diarrhea virus, recombinant protein, vaccine

## Abstract

**Background and Aim:**

Porcine epidemic diarrhea virus (PEDV) causes severe diarrhea in suckling piglets, leading to severe economic losses in the swine industry. Commercial vaccines have limited effectiveness against different genogroups of PEDV and the shedding of virus. The C-terminal of the S1 domain and the N-terminal of the S2 domain (S1-2) protein of the spike (S) protein have four neutralizing epitopes. However, research on the expression of the S1-2 segment of the S gene has been limited. In this study, we expressed a recombinant S1-2 protein of the S protein of the PEDV Thai isolate and characterized the immunological properties of the recombinant S1-2 protein.

**Materials and Methods:**

The S1-2 segment of the S gene of the PEDV Thai isolate (G2b) was amplified, cloned into the pBAD202/D-TOPO^®^ vector (Invitrogen, Carlsbad, CA, USA), and expressed in *Escherichia coli*. The optimum concentration of arabinose and the optimum induction time for the expression of the recombinant S1-2 protein were determined using sodium dodecyl sulfate-polyacrylamide gel electrophoresis. The immunogenic reactivity of the recombinant S1-2 protein was determined using Western blot analysis with rabbit polyclonal antibodies against the SM98 strain of PEDV (G1a).

**Results:**

The recombinant S1-2 segment of the S gene of the PEDV Thai isolate protein was cloned and the recombinant S1-2 protein was successfully expressed. The optimum concentration of arabinose and the optimum induction time for the induction of the recombinant S1-2 protein were 0.2% and 8 h, respectively. The recombinant S1-2 protein reacted specifically with both rabbit anti-histidine polyclonal antibodies and rabbit anti-PEDV polyclonal antibodies.

**Conclusion:**

The recombinant S1-2 protein reacted with rabbit anti-PEDV polyclonal antibodies induced by the different PEDV genogroup. Therefore, the recombinant S1-2 protein may be a useful tool for the development of a diagnostic test for PEDV or for a vaccine against PEDV.

## Introduction

Porcine epidemic diarrhea virus (PEDV) is a member of the genus *Alphacoronavirus*, family Coronaviridae. PEDV is an important causative agent of severe diarrhea in suckling piglets and results in severe economic losses to the swine industry [1]. PEDV is divided into genogroup 1 (G1) and genogroup 2 (G2) [2], based on the amino acid changes at the N-terminal domain (residues 21-324) of the S1 domain of the spike (S) protein [3]. G1 and G2 can, in turn, be subdivided into subgroups a and b. PEDV outbreaks have been reported worldwide since 1981 [1]. In Thailand, the first PEDV outbreak was reported in 2007 [4]. Recent Thai isolates have been shown to belong to both the G1 and G2 genogroups [5].

Vaccination is important in the control of porcine epidemic diarrhea. However, the efficacy of vaccines for PEDV is variable due to genetic differences between the vaccine strains and the field epidemic strains. Although the traditional vaccine has shown good efficacy against classical PEDV, a low efficacy of vaccine, characterized by high mortality rates of newborn piglets [1,6], is usually the result of using a vaccine strain that is genetically different from the field virus [7]. The S protein of PEDV has been shown to play an important role in stimulating neutralizing antibodies in the natural host. The S1 domain of the S protein has a specific role in binding with the host cell receptor [8]. The C-terminal of the S1 domain contains two epitopes, theCO-26K equivalent (COE) epitope located between amino acid residues 499 and 638, and a novel neutralizing epitope ^594^TSLLASACTIDLF GYP^609^ [9,10]. The COE epitope has been shown to be a core neutralizing epitope [11] and the C-terminus of COE (amino acid 575-639) has been shown to be a conformational epitope [12]. Even though the whole S1 domain might be suitable for immune response activation, the whole S1 domain protein has been shown to be the inducer of apoptosis in the host cells [13]. The N-terminal of the S2 domain (S1-2) of the S protein has two epitopes, ^753^YSNIGVCK^760^ and ^769^LQDGQVKI^776^ [14]. Several amino acid substitutions can be detected between field strains and vaccine strains at epitopes located on both the S1 (COE epitope and ^594^TSLLASACTIDL FGYP^609^) and S1-2 (^769^LQDGQVKI^776^). These amino acid substitutions (A522S, A554S, and G599S) involve serine substitution [15,16]. These changes may affect virus antigenicity and neutralizing activity [17].

The C-terminal part of the S1 domain and the N-terminal part of the S1-2 may counteract the apoptotic effects of the S1 domain. The S1-2 fragment of the S protein not only contains the COE (amino acids positions 499-638) and ^594^TSLLASACTIDLFGYP^609^ epitopes of the S1 domain but also contains the ^53^YSNIGV CK^760^ and ^769^LQDGQVK I^776^ epitopes of the S1-2. However, there have been few studies into the expression of this part of the S protein, with most work focusing only on the expression of the COE epitope.

Thus, the aims of the current study were to express the recombinant S1-2 fragment protein of the S protein of the PEDV Thai isolate using *Escherichia coli* expression system and to characterize the immunological properties of this recombinant protein.

## Materials and Methods

### Ethical approval

This study was approved by the Animal Ethics Committee of the Faculty of Veterinary Medicine, Kasetsart University, Bangkok, Thailand (ACKU60-VET-029).

### Study period and location

The study was conducted from July 2019 to November 2020 at the Faculty of Veterinary Medicine, Kasetsart University, Kamphaeng Saen Campus, Nakhon Pathom, Thailand.

### Samples

Small intestinal samples from diarrheic 5-day-old suckling pigs with suspected PEDV were collected and diagnosed using routine reverse transcription-polymerase chain reaction (RT-PCR) for PEDV at the Kamphaeng Saen Veterinary Diagnosis Center, Faculty of Veterinary Medicine, Kasetsart University. The PEDV-positive small intestinal samples were kept at −80°C until used.

### Amplification and cloning of the S1-2 segment of the PEDV S gene

Each 30 mg sample of a PEDV-positive small intestine was mixed with 200 μL of 1×phosphate-buffered saline (PBS, pH 7.4) and homogenized using a Bead Rupture 12 machine (Omni International, Kennesaw, GA, USA). RNA was extracted using a FavorPrep^™^ viral nucleic extraction Kit I (Favorgen, Ping-Tung, Taiwan) according to the manufacturer’s instructions. A set of primers were designed to amplify the S1-2 segment of the S gene: F (5′ CACC CTT CTG AGT CAC GAA CAG CCA 3′) and R (5′ GAG TCT CGC TTG AAC AGC ATA 3′). The CACC sequence at the 5′ end of the forward primer was used to clone the PCR products into the pBAD202/D-TOPO^®^ vector (Invitrogen, Carlsbad, CA, USA), according to manufacturer’s instructions. For cDNA synthesis, 20 μL of reverse transcription reaction mixture was used, comprising 4 μL of 5× reverse transcription buffer, 2 μL of random hexamer, 2 μL of 10 mM dNTP, 1 μL of MMLV reverse transcriptase (Thermo Fisher Scientific, Waltham, MA, USA), and 11 μL of the RNA template of the PEDV Thai isolate (G2b). The reverse transcription reaction conditions consisted of cDNA synthesis at 37°C for 1 h and enzyme inactivation at 70°C for 15 min. The PCR mixture was composed of 10 μL of 5× Phusion High-Fidelity buffer, 1 μL of 10 mM dNTP, 1 μL each of forward and reverse primers, 0.5 μL (2 U/mL) of Phusion Hot Start II DNA polymerase (Thermo Fisher Scientific), 5 μL of cDNA template, and 31.5 μL of distilled water, for a total volume of 50 μL. The PCR conditions were pre-denaturation at 95°C for 3 min, followed by 35 cycles at 95°C for 30 s, annealing at 56°C for 10 s, extension at 72°C for 30 s, and a final extension at 72°C for 10 min. The expected size of the PCR products was 1473 bp. The PCR products were analyzed using 1% agarose gel electrophoresis at 100 V for 30 min and visualized under ultraviolet illumination.

The PCR products of the S1-2 segment of the S gene were purified and cloned into plasmid pBAD202/D-TOPO^®^ (pBAD Directional TOPO^®^ expression vector; Invitrogen) according to manufacturer’s instructions. Briefly, 1 μL of purified PCR products was mixed with 1 μL of salt solution, 3 μL of sterile water, and 1μL of pBAD202/D-TOPO^®^ vector. The reaction mixture was mixed and incubated at 22°C for 30 min. Three microliters of the ligation mixture were used to transform into TOP10 competent cells (Invitrogen). Subsequently, 250 μL of super optimal broth with catabolite repression medium (pBAD Directional TOPO^®^ Expression Kit; Invitrogen) were added into the transformed TOP10 competent cells and shaken at 37°C for 1 h. Subsequently, transformed TOP10 competent cells (pBAD Directional TOPO^®^ Expression Kit; Invitrogen) were spread on Luria-Bertani (LB) agar containing 50 μg/mL of kanamycin. Positive colonies were confirmed using PCR, and the PCR products were sequenced at First BASE Co., Ltd. (Selangor, Malaysia). The deduced amino acid sequence of the S1-2 segment of the S gene was aligned with that of the S1-2 segment of the SM98 vaccine strain (G1a) using the BioEdit biological sequence alignment editor computer package (version 7.1.3; Ibis Biosciences, Carlsbad, CA, USA).

### Optimization for expression of the recombinant S1-2 fragment protein of the S gene

A single colony of recombinant *E. coli* was grown in 3 mL of LB broth containing 50 μg/mL of kanamycin, shaken overnight at 200 rpm at 37°C. Subsequently, 100 μL of starter culture were added into six tubes, each containing 10 mL of LB broth with 50 μg/mL of kanamycin, and were shaken at 200 rpm at 37°C until the optical density at 600 nm (OD_600_) reached 0.6. To determine the optimum arabinose concentration for the induction of the recombinant S1-2 fragment protein, 10-fold serial dilutions (final concentration of 0%, 0.00002%, 0.0002%, 0.002%, 0.02%, and 0.2%) of 20% arabinose were added to the LB broth and incubated at 37°C for 8 h. To determine the optimum induction time of the recombinant S1-2 fragment protein, 10 mL of the cultures were induced by 0.2% final concentration of arabinose, harvested every 2 h at 0, 2, 4, 6, and 8 h after induction, and kept at -80°C for further analysis using sodium dodecyl sulfate-polyacrylamide gel electrophoresis (SDS-PAGE). The wild-type TOP10 competent cells without the recombinant plasmids were used as a negative control. A 150 μg sample of each of the extracted proteins was loaded in each lane and analyzed using 10% SDS-PAGE for 45 min at 150 V. The amount of protein was measured using a Nanodrop 2000 Spectrophotometer (Thermo Fisher Scientific). Gels were stained with 0.01% Coomassie Brilliant Blue, 50% distilled water, 40% methanol, and 10% acetic acid for 15 min and destained with 50% distilled water, 40% methanol, and 10 % acetic acid. The expected size of the S1-2 fragment protein was 53 kDa. However, the molecular weight of the recombinant S1-2 protein would be 16 kDa larger than the expected size due to the thioredoxin and 6×histidine tag included in the recombinant protein. The recombinant S1-2 protein fragment was purified using HisTrap™ FF 1 mL (Ni-NTA column) with the AKTA system (GE Healthcare, Chicago, IL, USA) according to the manufacturer’s instructions.

### Western blot analysis of the recombinant S1-2 protein fragment

Purified recombinant S1-2 fragment proteins were separated using 10% SDS-polyacrylamide gels and electrotransferred onto nitrocellulose membranes using the semi-dry method with a Bio-Rad Tran-Blot SD Semi-Dry Electrophoretic Transfer Cell (Bio-Rad^®^, Hercules, CA, USA) at 10 V and 400 mA for 45 min. The nitrocellulose membranes were incubated with blocking buffer (5% skim milk in 1×PBS) at 37°C for 1 h. Subsequently, the membranes were incubated with either 1:5000 rabbit anti-histidine (GE Healthcare, Piscataway, NJ, USA) or 1:10 rabbit anti-PEDV polyclonal antibodies, produced by injecting rabbits with SM98 vaccine (ProVac^®^, Komipharm, GG, South Korea) 3 times at intervals of 2 weeks, and left overnight at 4°C. After washing with 1×PBS-T 3 times for 10 min, the membranes were incubated with 1:1000 goat anti-rabbit immunoglobulin G-horseradish peroxidase polyclonal antibodies (Invitrogen) at 37°C for 1 h. After washing, the membranes were incubated with diaminobenzidine (DAB) using a DAB substrate kit (Thermo Fisher Scientific) for 5-10 min at 25°C. The positive reaction was visualized as a dark brown band on the nitrocellulose membrane.

## Results

**Figure-1:**
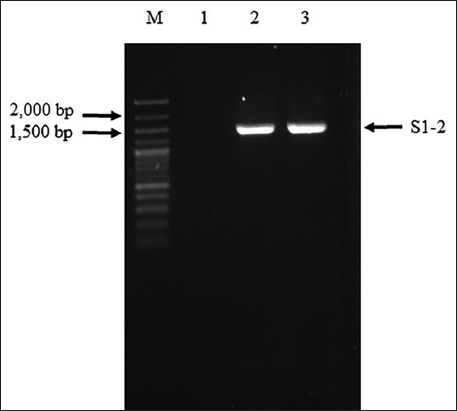
One percent agarose gel electrophoresis of polymerase chain reaction (PCR) products of S2 domain segment of spike gene of porcine epidemic diarrhea virus. Lane M=100 bp+3k DNA ladder (AccuBand™, SMOBIO Technology, Taiwan), Lane 1=negative control, lane 2=PCR products of positive sample (1473 bp), and lane 3=positive control.

**Figure-2:**
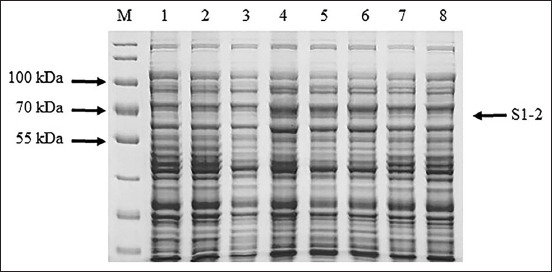
Ten percent sodium dodecyl sulfate-polyacrylamide gel electrophoresis analysis of optimum arabinose concentration for induction of recombinant S2 domain (S1-2) fragment protein expression. Lane M protein marker (PageRuler™ Plus Prestained Protein Ladder, Thermo Fisher Scientific, USA); lanes 1 and 2=proteins from wild type Top 10 competent cells induced with 0.2% arabinose for 0 and 8 h, respectively, and lanes 3-8=proteins from recombinant Top 10 competent cells containing S1-2 segment of porcine epidemic diarrhea virus S gene induced by 0, 0.2, 0.02, 0.002, 0.0002, and 0.00002% arabinose at 37°C for 8 h, respectively.

**Figure-3:**
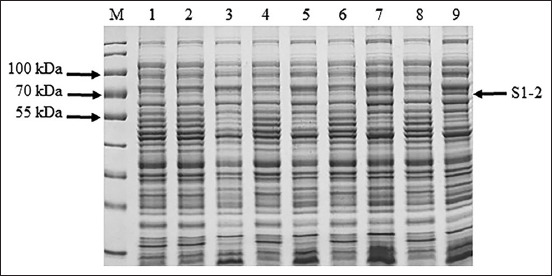
Ten percent sodium dodecyl sulfate-polyacrylamide gel electrophoresis analysis of optimum induction time for induction of recombinant S2 domain (S1-2) fragment protein expression using 0.2% arabinose. Lane M=protein marker (PageRuler™ Plus Prestained Protein Ladder, Thermo Fisher Scientific, USA); lanes 2, 4, 6, and 8=proteins from wild-type Top 10 competent cells induced for 2, 4, 6, and 8 h, respectively, and lanes 1, 3, 5, 7, and 9=proteins from recombinant Top 10 competent cells containing S1-2 segment of porcine epidemic diarrhea virus S gene induced for 0, 2, 4, 6, and 8 h, respectively.

**Figure-4:**
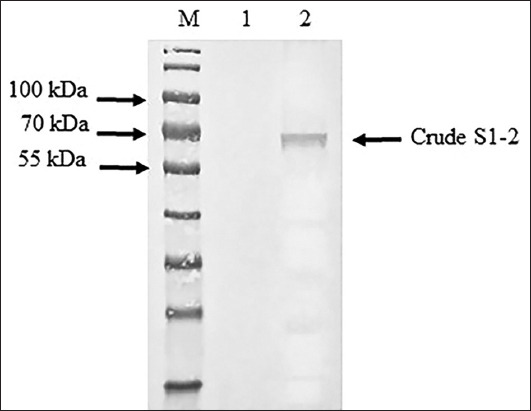
Western blot analysis of recombinant S2 domain (S1-2) fragment protein using rabbit anti-histidine polyantibodies. Lane M=protein marker (PageRuler™ Plus Prestained Protein Ladder, Thermo Fisher Scientific, USA); lane 1=crude protein from non-induced recombinant Top 10 competent cells containing S1-2 segment of porcine epidemic diarrhea virus (PEDV) S gene; and lane 2=crude protein from induced recombinant Top 10 competent cells containing S1-2 segment of PEDV S gene.

**Figure-5:**
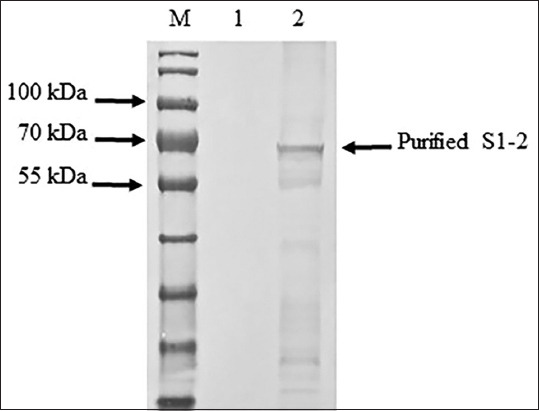
Western blot analysis of purified recombinant S2 domain (S1-2) fragment protein using rabbit anti-porcine epidemic diarrhea virus (PEDV) polyclonal antibodies. Lane M= protein marker (PageRuler™ Plus Prestained Protein Ladder, Thermo Fisher Scientific, USA); lane 1=purified protein of non-induced recombinant Top 10 competent cells containing S1-2 segment of PEDV S gene; and lane 2=purified of induced recombinant Top 10 competent cells containing S1-2 segment of PEDV S gene.

The S1-2 segment of the S gene of PEDV was successfully amplified and found to have a size of 1473 bp (Figure-1). The PCR products of the S1-2 fragment of the S gene were used to ligate the pBAD202/D-TOPO vector, and the recombinant plasmids were used to transform One Shot^®^ TOP10 competent cells (Thermo Fischer Scientific). Positive clones were confirmed using PCR, and the correctness of the inserted genes was confirmed by sequencing (accession number MW492057). The deduced amino acid of the S1-2 segment of the S gene showed the expected four epitopes: COE, ^594^TSLLASACTIDLFGYP^609^, ^753^YSNIGVCK^760^, and ^769^SQSSQVKI^776^. Amino acid alignment of the recombinant S1-2 fragment protein and the S1-2 fragment protein of the SM98 vaccine strain showed 28 amino acid substitutions, of which nine – L508S, A522S, L526R, S527G, V532I, T554S, G599S, A610E, and L617F – were at the COE; one, G599S, was in the ^594^TSLLASACTIDLFGY P^609^ epitope; and three – P769S, Y771S, and G772S – were in the ^769^LQDGQVKI^776^ epitope. However, the ^753^YSNIGVCK^760^ epitope was conserved between the recombinant S1-2 fragment protein and the S1-2 fragment of the SM98 vaccine strain. The optimum conditions for the expression of the recombinant S1-2 fragment protein of the S gene were 0.2% arabinose for 8 h (Figures-2 and 3). The molecular weight of the recombinant S1-2 fragment protein was approximately 69 kDa. Based on Western blot analysis, the crude and purified recombinant S1-2 fragment proteins reacted specifically with rabbit anti-histidine polyantibodies (Figure-4) and rabbit anti-PEDV polyclonal antibodies (Figure-5), respectively.

## Discussion

In this study, the S1-2 segment of PEDV was successfully cloned and expressed. The optimum conditions for the expression of the recombinant S1-2 fragment protein used here were 0.2% arabinose for 8 h. The molecular weight of the recombinant S1-2 fragment protein, which comprises amino acids 494-984 of the S gene, was approximately 69 kDa, which was 16 kDa higher than the expected size, due to the thioredoxin and 6×histidine included in the recombinant S1-2 protein fragment. However, information about the expression of the S1-2 segment of the S gene is very limited. Of the neutralizing epitopes in this segment of the S gene, only the COE epitope has been studied in any detail. The successful expression of the COE recombinant protein has been reported in several expression systems, including *E. coli* [9,18,19], transgenic tobacco [20], *Pichia pastoris* [21]*, Lactobacillus casei* [22], and mammalian cells [23]. The recombinant proteins of the COE region (amino acids 499-638) did not have apoptosis-inducing activity, as seen in the recombinant whole S1 domain of the S gene [9,18,19]. One study reported the expression of the recombinant protein of the C-terminal of the S1 domain and the N-terminal of the S1-2 (amino acid 482-840) in *E. coli* [11]. The recombinant protein in that study was shown to react with serum from naturally infected pigs and was shown to have neutralizing activity.

Western blot analysis indicated that the recombinant S1-2 fragment protein reacted specifically with rabbit anti-PEDV polyclonal antibodies. These polyclonal antibodies were induced by injecting rabbits with the SM98 vaccine strain (G1a) that had 28 amino acid differences from the recombinant S1-2 fragment protein in the current study, especially at the COE, the ^594^TSLLASACTIDLFGYP^609^ and the ^769^LQDGQVKI^776^ epitopes. This result was in keeping with that of a study that reported the recombinant protein of this region (amino acids 482-840) expressed in *E. coli* reacted with serum from naturally infected pigs [11]. The ability of the recombinant S1-2 fragment protein of PEDV (G2b) in the current study to react with antibodies induced by PEDV (G1a) showed the potential value of the recombinant S1-2 fragment protein for the detection of PEDV infection or for inducing the production of antibodies against different PEDV genogroups. Other reports have shown that the G1 and G2 genogroups induced by the whole recombinant S protein of each genogroup had different neutralizing activities [7,11]. However, further study is needed into the ability of the recombinant S1-2 protein fragment to induce neutralizing antibodies against PEDV or to detect antibodies against PEDV in either the same or a different genogroup.

## Conclusion

The recombinant S1-2 fragment protein of the PEDV Thai isolate could be expressed in *E. coli* and reacted specifically with rabbit anti-PEDV polyclonal antibodies induced by a different PEDV genogroup. *E. coli* expression system has many advantages: It is inexpensive, easy to manipulate, and produces a large amount of recombinant protein. Further study should investigate the potential use of the recombinant 1-2 fragment of the S protein for the development of a vaccine or diagnostic tools for PEDV.

## Authors’ Contributions

JS, NI, and TS: Designed and conducted the study and wrote the manuscript. NI, SJ, SP, KS, KaS, PL, and TS: Participated in the scientific discussion and supervised the study. All authors have read and approved the final manuscript.
